# Probing the Potential Energy Profile of the I + (H_2_O)_3_ → HI + (H_2_O)_2_OH Forward and Reverse Reactions: High Level CCSD(T) Studies with Spin-Orbit Coupling Included

**DOI:** 10.3390/molecules28020904

**Published:** 2023-01-16

**Authors:** Xinyuan Zhang, Xiaoting Chen, Yan Lin, Yan Meng, Guoliang Li, Yaoming Xie, Henry F. Schaefer

**Affiliations:** 1 Key Laboratory of Theoretical Chemistry of Environment, Ministry of Education, School of Chemistry, South China Normal University, Guangzhou 510006, China; 2 Center for Computational Quantum Chemistry, University of Georgia, Athens, GA 30602, USA

**Keywords:** iodine atom, water trimer, atom–molecule reactions, potential energy profile, CCSD(T) computations

## Abstract

Three different pathways for the atomic iodine plus water trimer reaction I + (H_2_O)_3_ → HI + (H_2_O)_2_OH were preliminarily examined by the DFT-MPW1K method. Related to previous predictions for the F/Cl/Br + (H_2_O)_3_ reactions, three pathways for the I + (H_2_O)_3_ reaction are linked in terms of geometry and energetics. To legitimize the results, the “gold standard” CCSD(T) method was employed to investigate the lowest-lying pathway with the correlation-consistent polarized valence basis set up to cc-pVQZ(-PP). According to the CCSD(T)/cc-pVQZ(-PP)//CCSD(T)/cc-pVTZ(-PP) results, the I + (H_2_O)_3_ → HI + (H_2_O)_2_OH reaction is predicted to be endothermic by 47.0 kcal mol^−1^. The submerged transition state is predicted to lie 43.7 kcal mol^−1^ above the separated reactants. The I···(H_2_O)_3_ entrance complex lies below the separated reactants by 4.1 kcal mol^−1^, and spin-orbit coupling has a significant impact on this dissociation energy. The HI···(H_2_O)_2_OH exit complex is bound by 4.3 kcal mol^−1^ in relation to the separated products. Compared with simpler I + (H_2_O)_2_ and I + H_2_O reactions, the I + (H_2_O)_3_ reaction is energetically between them in general. It is speculated that the reaction between the iodine atom and the larger water clusters may be energetically analogous to the I + (H_2_O)_3_ reaction. The iodine reaction I + (H_2_O)_3_ is connected with the analogous valence isoelectronic bromine/chlorine reactions Br/Cl + (H_2_O)_3_ but much different from the F + (H_2_O)_3_ reaction. Significant difference with other halogen systems, especially for barrier heights, are seen for the iodine systems.

## 1. Introduction

Iodine plays important roles in atmospheric and environmental chemistry [1,2]. An iodine atom can deplete tropospheric ozone via I + O_3_ → IO + O_2_, exacerbating the ozone hole in the lower stratospheric zone [3,4,5,6]. The forward and reverse reactions of the iodine atom plus water molecule have been the topic of various studies. This is because hydrogen iodide (HI) may be considered as a reservoir of chemically active iodine atoms in the atmosphere. As such, it can regenerate iodine atoms through its reaction with hydroxyl radicals [7,8,9,10,11,12,13]. Studies of the mechanism for the iodine plus water reaction are also important for the kinetics for severe light water reactor accidents [12,14], where the volatile iodine may be released from fuels and react with steam and hydrogen. Following previous studies of the iodine plus water monomer and dimer reactions [13,15], we expand our research to the iodine plus water trimer reaction, I + (H_2_O)_3_ → HI + (H_2_O)_2_OH, which is a better model to approach the reaction of iodine with water steam. We also compare the I + (H_2_O)_3_ reaction with the valence isoelectronic F/Cl/Br + (H_2_O)_3_ reactions [16,17,18] and discuss the main differences between them.

## 2. Results and Discussion

From previous research [19,20,21,22,23,24,25], the water trimer (H_2_O)_3_ has several isomers, of which the lowest energy isomer is *uud*-(H_2_O)_3_. Structurally, the *uud*-(H_2_O)_3_ has a six-membered ring structure consisting of three OH bonds (from three different water molecules) joined by three hydrogen bonds, with each OH serving as both electron donor and receptor. The orientations of the three out-of-ring OH bonds in *uud*-(H_2_O)_3_ are “up-up-down” (*uud*), with respective to the pseudoplanar six-membered ring.

Three different kinds of reaction pathways are predicted when an iodine atom approaches the water trimer (H_2_O)_3_ from different directions, as the MPW1K/cc-pVTZ(-PP) results show in Figure S1 in the Supplementary Materials. These pathways are similar in both energetics and structures, just as those for the F/Cl/Br + (H_2_O)_3_ reactions. To obtain more reliable predictions, the CCSD(T) method was adopted to further investigate the lowest-lying pathway, with basis sets up to cc-pVTZ(-PP) for geometry optimizations and vibrational frequency analyses and cc-pVQZ(-PP) for single-point energy computations. Thus, the following discussions are based on CCSD(T)/cc-pVTZ(-PP) geometries and vibrational frequencies and the CCSD(T)/cc-pVQZ(-PP)//CCSD(T)/cc-pVTZ(-PP) energetics, unless otherwise specified.

**Figure 1:**
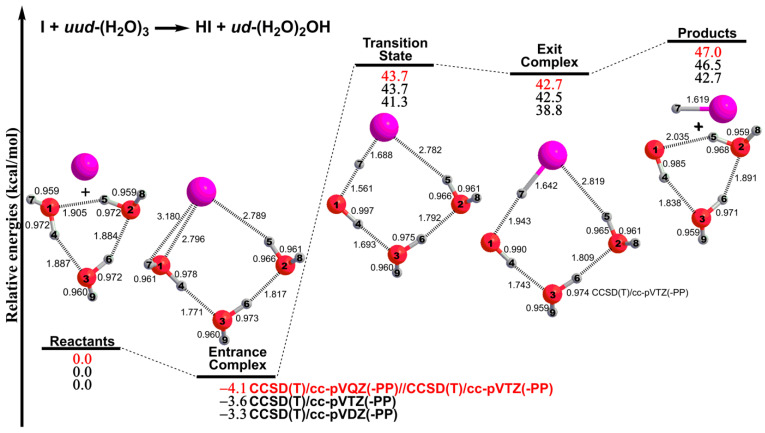
Stationary points on the lowest-energy potential energy profile of the I + (H_2_O)_3_ reaction. The distances and relative energies are given in angstroms and kcal mol^−1^, respectively.

Figure 1 shows that the I + (H_2_O)_3_ reaction starts with the formation of the entrance complex I∙∙∙(H_2_O)_3_, in which the I atom is bound to one water molecule, with the other two water molecules loosely linked. The I∙∙∙(H_2_O)_3_ entrance complex is predicted to lie 4.1 kcal mol^−1^ below the separated I + *uud*-(H_2_O)_3_ reactants.

In the transition state (TS), the forming I–H7 distance is decreased to 1.688 Å, much shorter than that for the I∙∙∙(H_2_O)_3_ entrance complex (3.180 Å), leading to the formation of an eight-membered ring structure containing three conventional OH bonds, one HI bond, three O∙∙∙H hydrogen bonds and one I∙∙∙H noncovalent interaction (Figure 1). The TS structure has an imaginary vibrational frequency of 307*i* cm^−1^ (as shown in Table S1 in the Supplementary Materials), with its normal mode corresponding to simultaneous O1–H7 elongation and I–H7 formation. The energy of the TS is 43.7 kcal mol^−1^ higher than that of the separated I + *uud*-(H_2_O)_3_ reactants.

The exit complex *ud*-HI∙∙∙(H_2_O)_2_OH also has an eight-membered ring structure, with its two out-of-plane OH moieties in “up-down” orientations. The *ud*-HI∙∙∙(H_2_O)_2_OH complex is very similar to the TS, differing mainly by the even longer O1–H7 and shorter I–H7 distance (Figure 1). The covalent I–H7 bond of 1.642 Å in the exit complex *ud*-HI∙∙∙(H_2_O)_2_OH is only slightly longer than the 1.619 Å in the free HI molecule. The exit complex *ud*-HI∙∙∙(H_2_O)_2_OH lies 42.7 kcal mol^−1^ energetically above the separated I + *uud*-(H_2_O)_3_ reactants but 4.3 kcal mol^−1^ below the separated HI + (H_2_O)_2_OH products.

Separating HI from the *ud*-HI∙∙∙(H_2_O)_2_OH exit complex leads to the reaction products HI and *ud*-(H_2_O)_2_OH. The two out-of-plane OH bonds of *ud*-(H_2_O)_2_OH are in the ‘‘up-down’’ orientations with respect to the pseudo six-membered ring plane. Compared with separated I + *uud*-(H_2_O)_3_ reactants, the HI + *ud*-(H_2_O)_2_OH products lie 47.0 kcal mol^−1^ above. Thus, the I + (H_2_O)_3_ → HI + (H_2_O)_2_OH reaction is significantly endothermic.

For the possible chemistry applications of this PES, we also considered the relative Gibbs free energies for the lowest-energy pathway of the I + (H_2_O)_3_ → HI + (H_2_O)_2_OH reaction at various conditions, as shown in Table S2. It appears that the different temperatures and pressures have little effect on the relative Gibbs free energies.

The harmonic vibrational frequencies and zero-point energies (ZPE) for the stationary points of the I + (H_2_O)_3_ → HI + (H_2_O)_2_OH reaction using the CCSD(T)/cc-pVTZ(-PP) method are shown in Table S1 of the Supplementary Materials. As seen from Table S1, our computational frequencies for (H_2_O)_3_ and (H_2_O)_2_OH agree with existing experimental values [26,27,28]. Especially, our theoretical H–I stretching frequency of 2314 cm^−1^ is very close to the experimental frequency of 2309 cm^−1^ [29]. The ZPE values given in Table S1 can be used to correct the energies of the stationary points. Including the ZPE corrections, the relative energies of the entrance complex, TS, exit complex and products for the I + (H_2_O)_3_ → HI + (H_2_O)_2_OH reaction become −4.1, 38.9, 38.3 and 41.7 kcal mol^−1^, respectively.

Spin-orbit coupling (SOC) effects also need to be considered in iodine-containing systems. In this research, the Breit–Pauli operator implemented in the MOLPRO program package is employed to provide SOC corrections, starting with the full valence complete active space self-consistent field (CASSCF) wave functions, using cc-pVQZ(-PP) basis sets [30,31]. For the reactant (I atom), entrance complex, TS, exit complex, and product [(H_2_O)_2_OH] in the lowest-energy pathways of the I + (H_2_O)_3_ reaction (Figure 1), our CASSCF SOC corrections are predicted to be 2425, 1229, 0.6, 0.1 and 4 cm^−1^ (or 6.9, 3.5, 0.0, 0.0, and 0.0 kcal mol^−1^), respectively. The SOC correction of 2425 cm^−1^ obtained herein for the I(^2^P) atom is in reasonable agreement with the experimental value of 2534 (= 7603/3) cm^−1^ [32]. With both ZPE and SOC corrections, the relative energies of the entrance complex, TS, exit complex and products for the I + (H_2_O)_3_ → HI + (H_2_O)_2_OH reaction become −0.7, 45.8, 45.2 and 48.6 kcal mol^−1^, respectively.

**Figure 2:**
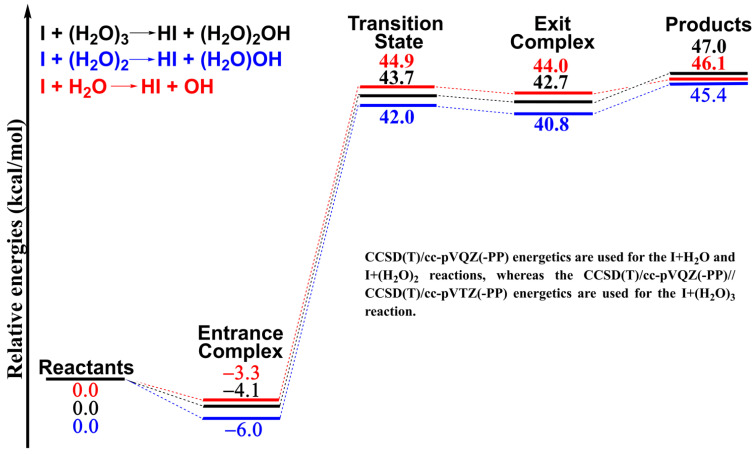
Comparison of the potential energy profiles for the I + (H_2_O)_3_, I + (H_2_O)_2_ and I + H_2_O reactions.

Next, we compare the water trimer reaction I + (H_2_O)_3_ with the water dimer reaction I + (H_2_O)_2_ and the water monomer reaction I + H_2_O. Structurally, the entrance complexes I∙∙∙(H_2_O)_3_, I∙∙∙(H_2_O)_2_ and I∙∙∙H_2_O are of some similarity [13,15]. The water trimer complex I∙∙∙(H_2_O)_3_ can be seen as the water dimer complex I∙∙∙(H_2_O)_2_ inserted into a third water molecule or having the water monomer complex I∙∙∙H_2_O associated with a water dimer. Similar cases occur for the transition states and the exit complexes. Energetically, the potential energy surfaces of the I + (H_2_O)_3_, I + (H_2_O)_2_ and I + H_2_O reactions are related, as shown in Figure 2. The trimer complex I∙∙∙(H_2_O)_3_ is bound by 4.1 kcal mol^−1^ (two new noncovalent interactions form but one is broken) lower than the binding energy of 6.0 kcal mol^−1^ (two new noncovalent interactions form) for the water dimer complex I∙∙∙(H_2_O)_2_ but higher than that of 3.3 kcal mol^−1^ (one new noncovalent interaction forms) for the water monomer complex I∙∙∙H_2_O. At the transition state, the relative energy of 43.7 kcal mol^−1^ for the water trimer reaction I + (H_2_O)_3_ is between those for the water dimer reaction (42.0 kcal mol^−1^) and water monomer reaction (44.9 kcal mol^−1^). A similar case occurs for the exit complex. For the separated products, the relative energy for the water trimer reaction (47.0 kcal mol^−1^) is somewhat higher than that for the water dimer reaction (45.4 kcal mol^−1^) and that for the water monomer reaction (46.1 kcal mol^−1^). With some exceptions, the water trimer reaction I + (H_2_O)_3_ looks energetically between the water dimer reaction I + (H_2_O)_2_ and the water monomer reaction I + H_2_O.

**Figure 3:**
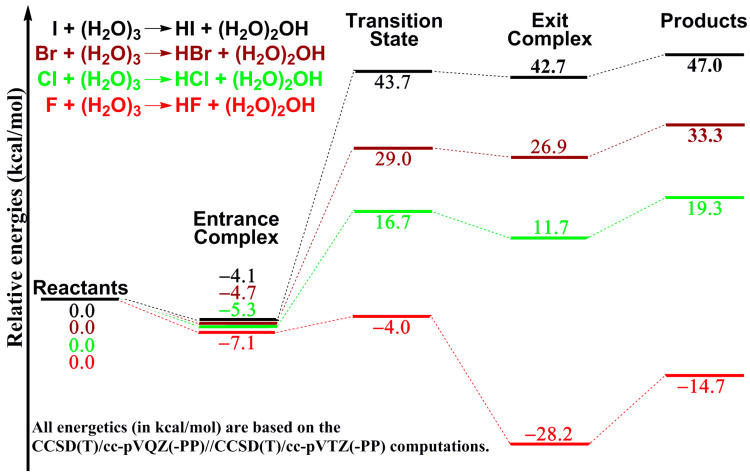
Comparison of the potential energy profiles for the I + (H_2_O)_3_ (black), Br + (H_2_O)_3_ (brown), Cl + (H_2_O)_3_ (green) and F + (H_2_O)_3_ (red) reactions. The PP is only used for the bromine and iodine atoms.

We also compare the potential energy profile of the I + (H_2_O)_3_ reaction with those for the Br + (H_2_O)_3_, Cl + (H_2_O)_3_ and F + (H_2_O)_3_ reactions. All five stationary points for the four reactions are geometrically related [16,17,18], while the landscape profiles of the four reactions are quantitatively different, as shown in Figure 3. The entrance well for I∙∙∙(H_2_O)_3_ lies below the reactants by 4.1 kcal mol^−1^, slightly shallower than the 4.7 kcal mol^−1^ for bromine, 5.3 kcal mol^−1^ for chlorine, and 7.1 kcal mol^−1^ for fluorine. The relative energies of the other stationary points (i.e., the transition state, exit complex and products) display significant differences. Taking the transition state as an example, the relative energy of 43.7 kcal mol^−1^ for the I + (H_2_O)_3_ reaction is much higher than that of 29.0 kcal mol^−1^ for the Br + (H_2_O)_3_ reaction. The analogous barriers are 16.7 kcal mol^−1^ for the Cl + (H_2_O)_3_ reaction and −4.0 kcal mol^−1^ for the F + (H_2_O)_3_ reaction. The endothermic energy decreases from 47.0 kcal mol^−1^ for the I + (H_2_O)_3_ reaction to 33.3 kcal mol^−1^ for the Br + (H_2_O)_3_ reaction and to 19.3 kcal mol^−1^ for the Cl + (H_2_O)_3_ reaction, while it is exothermic (by −14.7 kcal mol^−1^) for the F + (H_2_O)_3_ reaction. These energy differences should be related to the bond energy order of H–I (3.05 eV) < H–Br (3.76 eV) < H–Cl (4.43 eV) < H–F (5.87 eV) [29].

## 3. Computational Methods

Our preliminary computational method employed in this research is similar to that successfully used for the water dimer reaction of I + (H_2_O)_2_ [15], namely MPW1K, a density functional theory (DFT) method constructed by Truhlar et al. [33] MPW1K gave the best predictions among 49 DFT functionals used for the related F + H_2_O reaction barrier [34]. Our more reliable theoretical results come from the “gold standard” CCSD(T), the coupled-cluster single and double substitution method with a perturbative treatment of triple excitations [35,36,37].

In conjunction with the MPW1K and CCSD(T) methods, the correlation-consistent polarized valence basis sets (cc-pVnZ) of Dunning et al. were used. For the hydrogen and oxygen atoms, the cc-pVnZ (n = D, T, Q) basis sets [38,39] were utilized. For the iodine atom, the Stuttgart–Cologne pseudopotential (PP) and the corresponding cc-pVnZ-PP (n = D, T, Q) basis sets [40] of Peterson et al. were employed. The PP method replaces 28 inner core electrons (1s^2^2s^2^2p^6^3s^2^3p^6^3d^10^) of the iodine atom with an effective core potential.

The low energy pathways of the I + (H_2_O)_3_ reaction were firstly investigated at the MPW1K/cc-pVTZ(-PP) level of theory, using the Gaussian 16 program suite [41]. All of the stationary points involved were fully optimized and characterized via harmonic vibrational frequency analyses. Intrinsic reaction coordinate (IRC) [42,43,44] analyses were also performed with this method to ascertain that the transition state connects the designated entrance and exit complexes.

For the lowest-energy pathway of the I + (H_2_O)_3_ reaction, the CCSD(T)/cc-pVnZ(-PP) (n = D, T, Q) computations were performed, using the CFOUR program [45]. This allowed us to enhance the reliabilities of the geometries, energies and vibrational frequencies of the stationary points involved. In all CCSD(T) computations, the 1s-like MO for oxygen and the 4s4p4d-like MOs for iodine were frozen, i.e., doubly occupied. Restricted Hartree–Fock orbitals were used for all closed shell systems, while unrestricted orbitals were employed for the open-shell species.

## 4. Conclusions

Low energy pathways of the I + (H_2_O)_3_ → HI + (H_2_O)_2_OH reaction were explored using the “gold standard” CCSD(T) method. The Dunning correlation-consistent basis sets as large as cc-pVTZ(-PP) are used for the geometry optimizations and vibrational frequency analyses and cc-pVQZ(-PP) for the single-point energy determinations. Based on our CCSD(T)/cc-pVQZ(-PP)//CCSD(T)/cc-pVTZ(-PP) computations, the I + (H_2_O)_3_ → HI + (H_2_O)_2_OH reaction is significantly endothermic by 47.0 kcal mol^−1^. The submerged (compared to products) transition state lies 43.7 kcal mol^−1^ above the separated reactants, indicating there is no energy needed for the reverse reaction. Including zero-point energy and spin-orbit coupling corrections, the relative energies of the entrance complex, transition state, exit complex and products for the I + (H_2_O)_3_ → HI + (H_2_O)_2_OH reaction are predicted to be #x2212;0.7, 45.8, 45.2, and 48.6 kcal mol^−1^, respectively.

Compared with the related water dimer/monomer reactions I + (H_2_O)_2_/H_2_O, the stationary points of the water trimer reaction I + (H_2_O)_3_ may be structurally regarded as those of the I + (H_2_O)_2_ reaction inserted into a third water molecule or as those of the I + H_2_O reaction associating with a water dimer. Energetically, the entrance complex, transition state and exit complex of the I + (H_2_O)_2_ reaction have lower energies than those of the I + H_2_O reaction, while those of the I + (H_2_O)_3_ reaction have higher energies than those of the I + (H_2_O)_2_ reaction. This indicates that the second water molecule lowers the barrier of the water monomer reaction, but the third water molecule has almost no effect on the barrier. Thus, it is plausible that larger water clusters may behave energetically like the water trimer when reacting with an iodine atom. Of course, more research is necessary to prove this prediction.

The comparison of the I + (H_2_O)_3_ reaction with the analogous Br/Cl/F + (H_2_O)_3_ reactions finds that the four reactions are significantly different energetically. The I/Br/Cl + (H_2_O)_3_ reactions are all endothermic, with the reaction energy decreasing from 47.0 kcal mol^−1^ for I + (H_2_O)_3_ to 33.3 kcal mol^−1^ for Br + (H_2_O)_3_, and to 19.3 kcal mol^−1^ for Cl + (H_2_O)_3_, while the F + (H_2_O)_3_ reaction is exothermic with a reaction energy of -14.7 kcal mol^−1^. These reaction energies may be related to the bond energy order H–I < H–Br < H–Cl < H–F.

## Data Availability

The data presented in this study are available upon request from the corresponding authors.

## Supplementary Materials

The following supporting information can be downloaded at: https://www.mdpi.com/article/10.3390/molecules28020904/s1, Figure S1: Three pathways of the water trimer reaction I + (H_2_O)_3_ → HI + (H_2_O)_2_OH with the MPW1K/cc-pVTZ(-PP) method; Table S1: Harmonic vibrational frequencies and zero-point energies for the stationary points of the I + (H_2_O)_3_ → HI + (H_2_O)_2_OH reaction obtained at the CCSD(T)/cc-pVTZ(-PP) level of theory; Table S2: Relative Gibbs free energies for all stationary points of the I + (H_2_O)_3_ → HI + (H_2_O)_2_OH reaction at various conditions; Complete Gaussian 16 reference.
