# Supplementary material for: Probing the Potential Energy Profile of the I + (H2O)3 → HI + (H2O)2OH Forward and Reverse Reactions: High Level CCSD(T) Studies with Spin-Orbit Coupling Included
Source: Molecules. 2023 Jan 16;28(2):904. doi: 10.3390/molecules28020904 (PMC9866029; doi:10.3390/molecules28020904)
Supplement: Supplementary file 1 [file molecules-28-00904-s001.zip › molecules-2129060-supplementary.pdf]

*Supplementary Materials*

# Probing the Potential Energy Profile of the I + $(H_2O)_3$ → HI + $(H_2O)_2OH$ Forward and Reverse Reactions: High Level CCSD(T) Studies with Spin-Orbit Coupling Included

**Xinyuan Zhang [1,†], Xiaoting Chen [1,†], Yan Lin [1], Yan Meng [1], Guoliang Li [1,*], Yaoming Xie [2] and Henry F. Schaefer III [2,*]**

[1] Key Laboratory of Theoretical Chemistry of Environment, Ministry of Education; School of Chemistry, South China Normal University, Guangzhou 510006, China
[2] Center for Computational Quantum Chemistry, University of Georgia, Athens, GA 30602, USA
* Correspondence: glli@scnu.edu.cn (G.L.); ccq@uga.edu (H.F.S.III)
† These authors contributed equally to this work.

**Figure S1.** Three pathways of the water trimer reaction I + $(H_2O)_3$ → HI + $(H_2O)_2OH$ with the MPW1K/cc-pVTZ(-PP) method.

**Table S1**. Harmonic vibrational frequencies and zero-point energies for the stationary points of the I + $(H_2O)_3$ → HI + $(H_2O)_2OH$ reaction obtained at the CCSD(T)/cc-pVTZ(-PP) level of theory.

**Table S2.** Relative Gibbs free energies for all stationary points of the I + $(H_2O)_3$ → HI + $(H_2O)_2OH$ reaction at various conditions.

Complete Gaussian 16 reference.

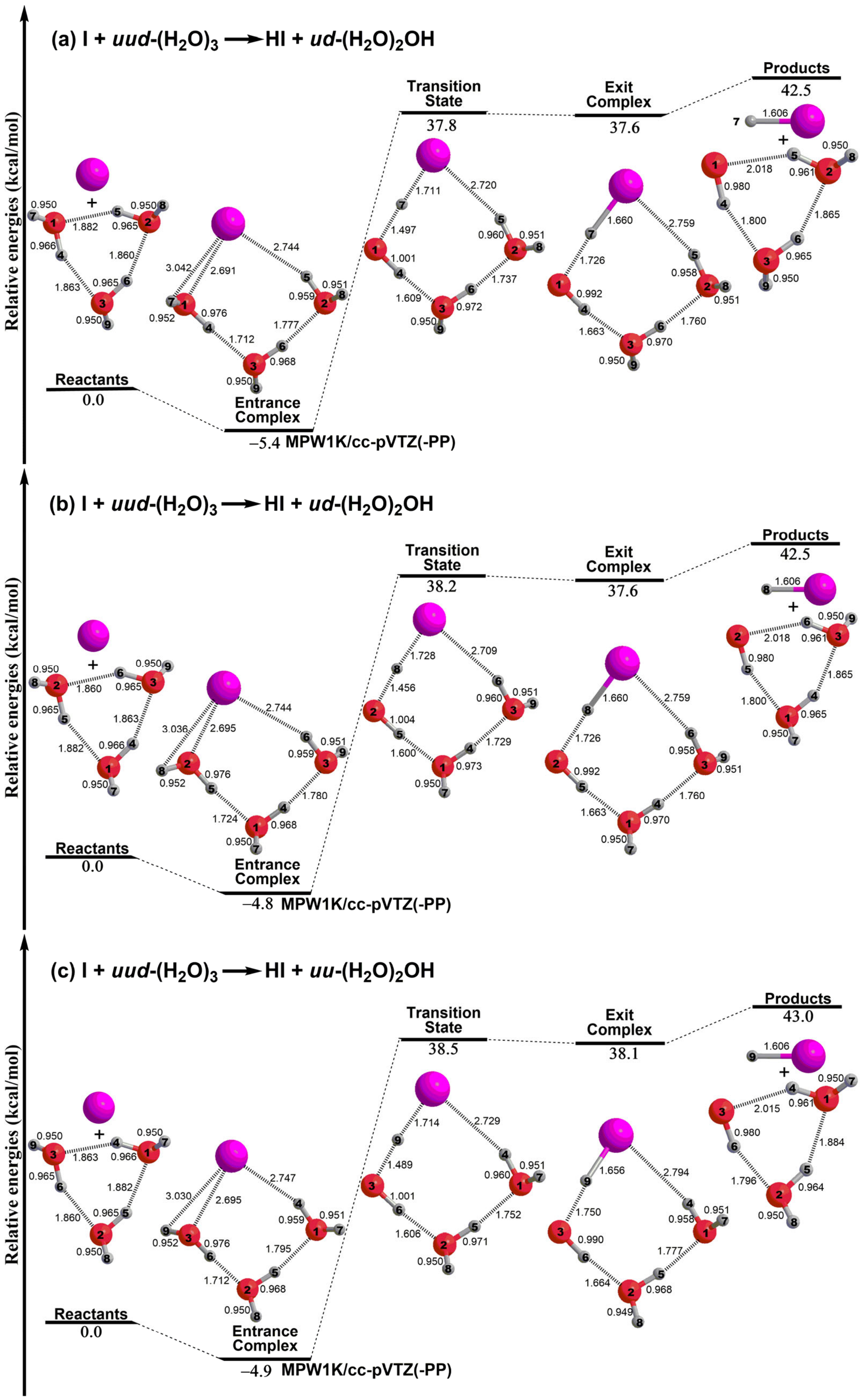


**Figure S1** Three pathways of the water trimer reaction I + $(H_2O)_3 \rightarrow$ HI + $(H_2O)_2OH$ with the MPW1K/cc-pVTZ(-PP) method. All bond distances were given in angstroms.

**Table S1**. Harmonic vibrational frequencies (in $cm^{-1}$) and zero-point energies (ZPE, in kcal/mol) for the stationary points of the I + $(H_2O)_3$ → HI + $(H_2O)_2OH$ reaction obtained at the CCSD(T)/cc-pVTZ(-PP) level of theory. Experimental results are also shown for comparison.

| | ZPE | ΔZPE | Vibrational Frequencies |
|---|---|---|---|
| $(H_2O)_3$ | 46.46 | 0.00 | 188, 194, 202, 209, 228, 253, 360, 372, 471, 614, 689, 926, 1685, 1690, 1707, 3613, 3683, 3689, 3903, 3907, 3909 |
| Entrance Complex | 46.43 | -0.03 | 30, 53, 100, 151, 180, 220, 237, 250, 261, 343, 390, 445, 491, 755, 903, 1670, 1689, 1706, 3521, 3635, 3769, 3876, 3900, 3901 |
| Transition State | 41.66 | -4.80 | 307*i*, 30, 63, 101, 186, 214, 252, 277, 286, 406, 481, 493, 577, 689, 814, 1016, 1451, 1669, 1700, 3271, 3598, 3766, 3900, 3902 |
| Exit Complex | 42.05 | -4.41 | 28, 46, 95, 115, 167, 215, 240, 267, 270, 331, 366, 446, 480, 597, 758, 903, 1670, 1698, 2093, 3410, 3626, 3780, 3901, 3907 |
| $(H_2O)_2OH$ | 37.85 | -5.30 | 163, 196, 211, 230, 252, 291, 375, 534, 557, 668, 916, 1667, 1687, 3498, 3675, 3736, 3908, 3911 |
| HI | 3.31 | | 2314 |
| **Experiment** | | | |
| Bonded OH in $(H_2O)_3$ | | | 3533,[a] 3544/3529,[b] 3528,[c] 3531.8±1.2,[d] 3516.7±2.3[d] |
| Free OH in $(H_2O)_3$ | | | 3726,[a] 3717[b] |
| OH radical in $(H_2O)_2OH$ | | | 3365.2[c] |
| HI | | | 2309[e] |

[a] In gas phase from Ref. 26. [b] In liquid He from Ref. 26. [c] In solid Ne from Ref. 27.
[d] From Ref. 28. [e] From Ref. 29, which is a harmonic vibrational frequency.

**Table S2.** Relative Gibbs free energies (ΔG, in kcal/mol) for all stationary points of the I + $(H_2O)_3$ → HI + $(H_2O)_2OH$ reaction, as shown in Figure 1, at various conditions.[a]

| | T = 600 K<br>p = 160 atm | T = 298.15 K<br>p = 1.0 atm | T = 200 K<br>p = 0.2 atm |
|---|---|---|---|
| **I + $(H_2O)_3$** | 0.0 | 0.0 | 0.0 |
| **Entrance Complex** | 1.6 | 2.1 | 0.8 |
| **Transtion State** | 46.1 | 45.3 | 43.7 |
| **Exit Complex** | 42.1 | 43.4 | 42.3 |
| **HI + $(H_2O)_2OH$** | 37.1 | 39.3 | 40.0 |

[a] Total energies (E) were obtained at the CCSD(T)/cc-pVTZ(-PP) level of theory.

**Complete Gaussian 16 reference**

M. J. Frisch, G. W. Trucks, H. B. Schlegel, G. E. Scuseria, M. A. Robb, J. R. Cheeseman, G. Scalmani, V. Barone, G. A. Petersson, H. Nakatsuji, X. Li, M. Caricato, A. V. Marenich, J. Bloino, B. G. Janesko, R. Gomperts, B. Mennucci, H. P. Hratchian, J. V. Ortiz, A. F. Izmaylov, J. L. Sonnenberg, D. Williams-Young, F. Ding, F. Lipparini, F. Egidi, J. Goings, B. Peng, A. Petrone, T. Henderson, D. Ranasinghe, V. G. Zakrzewski, J. Gao, N. Rega, G. Zheng, W. Liang, M. Hada, M. Ehara, K. Toyota, R. Fukuda, J. Hasegawa, M. Ishida, T. Nakajima, Y. Honda, O. Kitao, H. Nakai, T. Vreven, K. Throssell, J. A. Montgomery, Jr., J. E. Peralta, F. Ogliaro, M. J. Bearpark, J. J. Heyd, E. N. Brothers, K. N. Kudin, V. N. Staroverov, T. A. Keith, R. Kobayashi, J. Normand, K. Raghavachari, A. P. Rendell, J. C. Burant, S. S. Iyengar, J. Tomasi, M. Cossi, J. M. Millam, M. Klene, C. Adamo, R. Cammi, J. W. Ochterski, R. L. Martin, K. Morokuma, O. Farkas, J. B. Foresman, and D. J. Fox, Gaussian 16, Revision B.01, Gaussian, Inc., Wallingford CT, **2016**.
